# Beyond Neurovascular Repair: Should Epileptogenesis Inform VEGF Therapy After Intracerebral Hemorrhage?

**DOI:** 10.31083/RN56449

**Published:** 2026-09-18

**Authors:** Alejandro Chapa-Rodriguez, Yasier Medina

**Affiliations:** ^1^Department of Pulmonary and Critical Care Medicine, Cape Fear Valley Medical Center, Fayetteville, NC 28304, USA; ^2^Department of Neurology, Cape Fear Valley Medical Center, Fayetteville, NC 28304, USA

To the Editor,

We read with interest the review by Niu et al. [1] on the dual role of vascular endothelial growth factor (VEGF) in intracerebral hemorrhage (ICH). The authors move beyond a binary framework by proposing phase-dependent effects: early VEGFR-2/Src-mediated blood-brain barrier (BBB) disruption followed, during the subacute and chronic phases, by VEGF-mediated angiogenesis, glymphatic restoration, and neurovascular repair. This temporal model is a useful conceptual advance and a sound structure for translational planning. Post-ICH seizures and epileptogenesis, however, are absent from the proposed framework.

This omission is notable because several mechanisms described in the review overlap with those implicated in post-injury epileptogenesis. In Section 7.1 (see also Fig. 2 and Table 1 of that review), the authors detail VEGFR-2/Src-mediated phosphorylation of VE-cadherin, disassembly of adherens junctions with internalization of claudin-5 and occludin, and matrix metalloproteinase-2 and matrix metalloproteinase-9 (MMP-2/MMP-9)-mediated degradation of the endothelial basement membrane [1,2]. The immediate consequence is extravasation of serum proteins into the neuropil. Experimental exposure of brain tissue to albumin causes astrocytic uptake through transforming growth factor-beta receptor signaling, downregulation of Kir4.1 channels, impaired extracellular potassium buffering, and epileptiform activity [3]. BBB dysfunction is also increasingly implicated in post-stroke epileptogenesis after both ischemic and hemorrhagic injury [4]. A biologically plausible delayed consequence of the cascade described by Niu et al. [1] may therefore be epileptogenic network remodeling, which is not addressed in the review.

This issue is particularly relevant to the biphasic therapeutic strategy proposed in Section 8.1.4. The authors advocate deliberate activation of VEGF-C/VEGFR-3 or VEGFR-2-mediated regenerative signaling after the acute phase. In Section 7.2 and Table 1 of that review, the subacute–chronic phase is identified as days 3–14, with Section 7.2 extending this phase beyond day 14. This interval overlaps with the latent period following an epileptogenic insult. In mice subjected to pilocarpine-induced status epilepticus, VEGFR-3 expression was significantly increased by day 4, while VEGF-C expression continued to rise and peaked at day 7 [5]. Thus, the proposed reparative window coincides with dynamic regulation of the same signaling axis during early network remodeling after status epilepticus. This temporal overlap does not establish that therapeutic VEGF-C/VEGFR-3 activation after ICH is pro-epileptogenic; rather, it supports including seizure outcomes in future studies. The argument for receptor selectivity does not fully resolve this concern. Niu et al. [1] propose VEGF-C/VEGFR-3 activation as the safer option because VEGFR-3 preferentially engages phosphoinositide 3-kinase/protein kinase B signaling pathway (PI3K/Akt) while avoiding the pro-permeability Src pathway. This distinction may be less absolute *in vivo*. Proteolytic processing converts VEGF-C into a mature form capable of binding and activating VEGFR-2 as well as VEGFR-3 [6], and VEGF-C can induce VEGFR-2/VEGFR-3 heterodimer formation on angiogenic sprouts [7]. Although these observations were not obtained in post-ICH brain, they caution against treating VEGFR-3 selectivity as absolute. Receptor-selective therapy may produce preferential rather than exclusive signaling, and concurrent VEGFR-2 activation cannot be assumed to be absent.

The direction of the effect remains unresolved. VEGF has demonstrated anticonvulsant effects on epileptiform activity in an experimental hippocampal model [8], whereas VEGF-C, VEGFR-2, and VEGFR-3 expression is elevated after experimental status epilepticus [5] and in resected tissue from patients with mesial temporal lobe epilepsy [9]. This upregulation could represent a pathophysiologic mechanism, an endogenous neuroprotective response, or both. Moreover, these findings derive from status epilepticus and mesial temporal lobe epilepsy, which are not equivalent to post-ICH epileptogenesis, and therefore do not establish that VEGF-C/VEGFR-3 signaling promotes post-ICH epilepsy. They do show that components of the VEGF-C/VEGFR-3 pathway are dynamically regulated following epileptogenic insults during the same interval in which therapeutic activation has been proposed.

Common experimental and early translational endpoints, including hematoma volume, perihematomal edema, and 90-day functional scales, may not detect a delayed effect on seizure risk. Among patients surviving the first week after ICH, late seizures occurred in 9.2%, with cumulative risk increasing from 7.1% at 1 year to 10.0% at 2 years and 11.8% at 5 years [10]. A therapy that improves early functional outcomes while altering delayed seizure risk could therefore appear successful under conventional trial designs.

We suggest that translational studies of VEGF modulation in ICH prespecify seizure-related outcomes. These should distinguish acute symptomatic seizures occurring within 7 days of ICH, late unprovoked seizures occurring after day 7, and post-ICH epilepsy as a distinct clinical outcome. A 24-month follow-up could therefore serve as a pragmatic minimum: a 12-month endpoint would capture roughly 60% of the events observed by five years, whereas 24 months would capture approximately 85% [10]. Late seizures could be identified through scheduled follow-up and review of interim clinical records, with suspected events reviewed to determine whether they met criteria for a late unprovoked seizure or post-ICH epilepsy.

During the acute hospitalization, continuous electroencephalography (EEG) monitoring for at least 24 hours could be prespecified when there is clinical suspicion of seizures or otherwise unexplained or fluctuating mental status, with longer or repeat monitoring during the subacute period when clinically indicated [11]. Electrographic seizures should be analyzed as seizure outcomes, whereas lateralized periodic discharges, lateralized rhythmic delta activity, and other epileptiform abnormalities should be analyzed separately as exploratory markers of cortical irritability or increased seizure risk rather than as evidence of epileptogenesis [12]. Analyses should also account for cortical involvement rather than relying only on broad lobar-versus-deep categories. The cortical involvement, age <65 years, hematoma volume >10 mL, and early seizure (CAVE) score assigns one point each for cortical involvement, age younger than 65 years, hematoma volume greater than 10 mL, and early seizures, and stratifies late-seizure risk from 0.6% to 46.2% across its range [10]. For VEGF-directed therapy initiated before completion of the first 7 days, the three baseline components (cortical involvement, age, and hematoma volume) could be used for initial risk assessment, with the complete CAVE score calculated after the early-seizure window has passed. For treatment initiated after day 7, the complete score would be available at baseline. These additions would help determine whether interventions optimized for neurovascular repair also alter long-term cortical excitability.

Niu et al. [1] have provided a valuable mechanistic synthesis, and their phase-dependent framework is well suited to the development of VEGF-directed therapy. Extending that framework to include electrical network remodeling would strengthen its translational relevance and help define not only when VEGF modulation may be beneficial, but also whether it alters long-term seizure risk.
